# Nanopore quality score resolution can be reduced with little effect on downstream analysis

**DOI:** 10.1093/bioadv/vbac054

**Published:** 2022-08-11

**Authors:** Martín Rivara-Espasandín, Lucía Balestrazzi, Guillermo Dufort y Álvarez, Idoia Ochoa, Gadiel Seroussi, Pablo Smircich, José Sotelo-Silveira, Álvaro Martín

**Affiliations:** Instituto de Computación, Facultad de Ingeniería, Universidad de la República, 11300 Montevideo, Uruguay; Departamento de Genética, Facultad de Medicina, Universidad de la República, 11800 Montevideo, Uruguay; Departamento de Genómica, Instituto de Investigaciones Biológicas Clemente Estable, 11600 Montevideo, Uruguay; Instituto de Computación, Facultad de Ingeniería, Universidad de la República, 11300 Montevideo, Uruguay; Sección Bioinformática, Unidad de Genómica Evolutiva, Facultad de Ciencias, Universidad de la República, 11400 Montevideo, Uruguay; Instituto de Computación, Facultad de Ingeniería, Universidad de la República, 11300 Montevideo, Uruguay; Electrical and Electronics Department, Tecnun, University of Navarra, 20018 San Sebastián, Spain; Electrical and Computer Engineering, University of Illinois at Urbana-Champaign, Champaign, IL 61801, USA; Instituto de Computación, Facultad de Ingeniería, Universidad de la República, 11300 Montevideo, Uruguay; Instituto de Ingeniería Eléctrica, Facultad de Ingeniería, Universidad de la República, 11300 Montevideo, Uruguay; Departamento de Genómica, Instituto de Investigaciones Biológicas Clemente Estable, 11600 Montevideo, Uruguay; Laboratorio de Interacciones Moleculares, Facultad de Ciencias, Universidad de la República, 11400 Montevideo, Uruguay; Departamento de Genómica, Instituto de Investigaciones Biológicas Clemente Estable, 11600 Montevideo, Uruguay; Departamento de Biología Celular y Molecular, Sección Biología Celular, Facultad de Ciencias, Universidad de la República, 11400 Montevideo, Uruguay; Instituto de Computación, Facultad de Ingeniería, Universidad de la República, 11300 Montevideo, Uruguay

## Abstract

**Motivation:**

The use of high precision for representing quality scores in nanopore sequencing data makes these scores hard to compress and, thus, responsible for most of the information stored in losslessly compressed FASTQ files. This motivates the investigation of the effect of quality score information loss on downstream analysis from nanopore sequencing FASTQ files.

**Results:**

We polished *de novo* assemblies for a mock microbial community and a human genome, and we called variants on a human genome. We repeated these experiments using various pipelines, under various coverage level scenarios and various quality score quantizers. In all cases, we found that the quantization of quality scores causes little difference (or even sometimes improves) on the results obtained with the original (non-quantized) data. This suggests that the precision that is currently used for nanopore quality scores may be unnecessarily high, and motivates the use of lossy compression algorithms for this kind of data. Moreover, we show that even a non-specialized compressor, such as gzip, yields large storage space savings after the quantization of quality scores.

**Availability and supplementary information:**

Quantizers are freely available for download at: https://github.com/mrivarauy/QS-Quantizer.

## 1 Introduction

The precision for quality score representation in FASTQ files has historically been relatively large, where each score takes a value within a set of more than 40 possible values. The precise definition of this set depends on the sequencing technology; sequencers from Oxford Nanopore Technologies (https://nanoporetech.com/) use a set of 94 different values (represented in FASTQ files as ASCII codes 33–126). This high precision makes quality score sequences hard to compress and, thus, most of the information stored in a losslessly compressed FASTQ files corresponds to quality scores (substantially more than the genomic information itself). With growing concerns about the economics of storage space and bandwidth for transmission of genomic data, this strategy is being questioned. For example, in the context of short-read sequencing technologies, Illumina has reduced the set of possible values for quality scores on their most modern equipment to just four different values (Ill, 2017) and suggests similarly quantizing the values produced by other Illumina devices (Ill, 2014). It has also been observed that it is possible to use lossy compression algorithms, in which the decompressed data may differ to some extent from the original, without generally affecting the results of commonly used pipelines (Cánovas *et al.*, 2014; Ochoa *et al.*, 2013, 2016).

Nanopore sequencing, however, is a much more recent technology and few specific data compressors suitable for nanopore data are available, developed by our group (Dufort y Álvarez *et al.*, 2020, 2021) and others (Kokot *et al.*, 2022; Meng *et al.*, 2021). Moreover, the lossy compression of quality scores for nanopore data has only been explored in (Kokot *et al.*, 2022), where the impact of quality score information loss is assessed for some downstream analyses. Specifically, in Kokot *et al.* (2022), it is shown that this information loss has no or little impact on the construction of consensus sequences with Racon (Vaser *et al.*, 2017) for long CHM13 reads, either for HiFi or for Nanopore data. The impact is also shown to be small for variant calling accuracy with HiFi reads, evaluated with DeepVariant (Poplin *et al.*, 2018) on HG002 HiFi reads against the human reference genome GRCh38; variant calling for nanopore data is not evaluated.

Adding additional new evidence to, and reinforcing the conclusions of Kokot *et al.* (2022), in this article, we evaluate the impact of nanopore quality score quantization on various downstream applications, by comparing the results obtained with certain non-quantized nanopore data sets, referred to as *original data*, and with quantized versions of the same data sets. Particularly we selected data sets relevant for microbiology and human genetics, both areas of high interest and increasing use of this sequencing technology. The quantizers that we evaluate are mostly simple functions, where the set of possible quality scores is partitioned into a small set of disjoint intervals, each of which is mapped to a fixed value. For example, one such quantizer maps quality scores between 0 and 7 to the fixed score 5, and all quality scores larger than 7 to the fixed score 15. We also evaluate a slightly more complex scheme, which uses higher quantization resolution for quality scores associated with repetitive regions of a read. A precise definition of all evaluated quantizers is given in Section 2.1. We point out that these quantizers have not been optimized for any specific application; as it turns out, these definitions suffice to obtain a performance very close to that obtained with non-quantized data for all tested applications. A more thorough optimization of the quantizers is likely to be application-dependent and is deferred to future research.

To assess the effect of quality score quantization on typical bioinformatic downstream analyses, we focused on frequently used Nanopore applications where quality scores have a role. Long reads are regularly used in genome assembly, but most assemblers do not use quality scores (Kolmogorov *et al.*, 2019; Koren *et al.*, 2017; Ruan and Li, 2020). Structural variation identification (Cretu Stancu *et al.*, 2017; Heller and Vingron, 2019; Sedlazeck *et al.*, 2018; Tham *et al.*, 2020) and variant calling (Edge and Bansal, 2019; Shafin *et al.*, 2021) are also among Nanopore most frequent research applications, but generally just the latter makes use of quality scores. Given the high error rates of Nanopore sequencing data, strategies involving polishing algorithms are almost mandatory, and they are sensitive to quality scores. Based on these points, we carried on assembly polishing for the ZymoBIOMICS Microbial Community Standard (Zymo Research Corporation, Irvine, CA, USA. Product D6300, Lot ZRC190633) nanopore reads using Racon (Vaser *et al.*, 2017) both alone and combined with Medaka (https://github.com/nanoporetech/medaka), and MarginPolish (Shafin *et al.*, 2020) both alone and combined with HELEN (Shafin *et al.*, 2020). We also evaluated the quality of human genome assembly from nanopore reads polished with MarginPolish both alone and combined with HELEN, for several coverage levels. For variant calling, we executed PEPPER-Margin-DeepVariant (Shafin *et al.*, 2021) on sample HG003 for several coverage level scenarios. Details on the methods for evaluating assembly polishing and variant calling pipelines are presented in Sections 2.2 and 2.3, respectively.

As shown in Section 3, all the experimented pipelines yield very similar performance on the original and quantized versions of each data set, indicating that the effect of information loss caused by quality score quantization is not significant in practice. For the assembly polishing of a mock microbial community, setting all quality scores to the fixed value 10 results in a number of mismatches that is, on average over three independent runs, <1.2% higher that obtained with the original data. A quantizer that uses four different values for quality scores results in a number of mismatches that is, on average, even smaller than that obtained with the non-quantized data. This same quantizer, applied to human data, results in a number of mismatches that is essentially equivalent to that obtained with the original data; even for a very low coverage (10% of the original reads), the increment in the number of mismatches is only about 0.7%. Moreover, we show that the quantization of quality scores yields very large improvements in compression performance even for traditional non-specialized compressors such as gzip (see Sections 2.4 and 3). For example, we report on human variant calling results at 90× coverage where using 8 different values for quality scores yields precision and recall metrics for single-nucleotide polymorphisms (SNPs) that differ by <$10^{-4}$ from those obtained with the original (non-quantized) data set, while saving more than 33% of the required storage space. Even using only two different values for quality scores, which saves almost 70% of storage space, the precision differs by <$10^{-4}$ and the recall by <$10^{-3}$ from those obtained with the original data set.

## 2 Methods

### 2.1 Quantization of quality scores

We tested various quantizers, each mapping quality scores to values on a (small) set referred to as the *quantization alphabet*. We denote by *Q_i_* a quantizer for a quantization alphabet of size *i*, *i *>* *1, where the quantization $Q_{i}(x)$ of a quality score *x* depends solely on *x*. The specific definitions of *Q*_2_, *Q*_4_ and *Q*_8_ are presented in Table 1. All these quantizers collapse a large set of high-quality scores into a single value, and define a finer partition for lower scores, which occur more frequently (Delahaye and Nicolas, 2021). Our experiments suggest that slight variations of quantization level thresholds and quantization alphabet in the definitions of these quantizers have little effect on their performance. For example, modifying *Q*_2_ so that the values in {0…6} are mapped to 1, and those in {7…93} are mapped to 13, produces a variation of only 0.13% on the resulting F1 score for SNPs variant calling at 30× coverage (see Section 2.3). We also tested constant quantizers, which map every quality score to a fixed prescribed value. We denote by *F_z_* a quantizer that assigns the value $F_{z}(x)=z$ to every quality score *x*. In our experiments, we take *z *=* *10, which is a common threshold for filtering low-quality reads (see, e.g. Shafin *et al.*, 2021).

**Table 1.** Definition of quantizers *Q*_2_, *Q*_4_ and *Q*_8_

Quantizer	Quality scores	Quantized value
*Q*_2_	0…7	5
	8…93	15
*Q*_4_	0…7	5
	8…13	12
	14…19	18
	20…93	24
*Q*_8_	0…6	5
	7…11	10
	12…16	15
	17…21	20
	22…26	25
	27…31	30
	32…36	35
	37…93	40

In addition, since repetitive patterns of bases are particularly difficult to sequence by nanopore technologies (Delahaye and Nicolas, 2021), we evaluate quantizers where the quantization of a quality score *x* in a position *j* of a read depends not only on *x* but also on the bases called in positions close to *j*. We say that a substring of the base call sequence of a read is a *repetitive sequence* if it is comprised of either *h* consecutive copies of the same symbol, i.e. an homopolymer of length *h*, or *d* consecutive copies of the same pair of symbols, i.e. a repeat of *d* dinucleotides. For two quantization functions, *f*, f′, we denote by ${\langle f,f\prime \rangle }_{\delta }$ a quantizer that quantizes *x* to *f*(*x*), if the position *j* of the quality score *x* in a read is more than *δ* bases away from a repetitive sequence in this read, and to f′(x) otherwise. For example, the quantizer ${\langle F_{10},Q_{8}\rangle }_{\delta }$ sets all quality scores away from repetitive regions to the fixed value 10, and applies *Q*_8_ to quality scores that are within or close to repetitive regions. For the experiments reported in Section 3, the parameters *h*, *d* and *δ* are set to 5, 4 and 5, respectively. Using the surrounding bases to select an appropriate quantizer resembles previous works on quality score compression for short-read technologies, where the quantization granularity for all quality scores mapped to a given locus is selected based on the bases mapped at that position (Voges *et al.*, 2018). As they showed in Voges *et al.* (2018) and we show in Section 3, using the information from the bases to select the quantization level has no negative effect on the subsequent downstream analyses, which further suggests that the bases provide insightful information to guide the selection of distortion level for the quality values.

### 2.2 Assembly polishing

We evaluated the impact of quality score quantization on the genome assembly polishing for the ZymoBIOMICS Microbial Community Standard (Zymo Research Corporation, Irvine, CA, USA. Product D6300, Lot ZRC190633). For the experiments, we used the data generated by the authors of (Nicholls *et al.*, 2019) on Oxford Nanopore GridION, R10.3 pore (https://nanopore.s3.climb.ac.uk/mock/Zymo-GridION-EVEN-3Peaks-R103-merged.fq.gz). The organisms in this community are 8 bacteria, each present at 12%, and 2 yeasts, each present at 2%. We assembled the genomes with Flye (Kolmogorov *et al.*, 2020) and kept the eight largest contigs, which correspond to the eight bacteria. We polished this raw assembly using various polishing pipelines:

- *Racon*: 2 iterations of Racon (Vaser *et al.*, 2017).
- *Medaka*: 2 iterations of Racon followed by Medaka (https://github.com/nanoporetech/medaka).
- *MP*: An execution of MarginPolish (Shafin *et al.*, 2020).
- *Helen*: An execution of MarginPolish followed by HELEN (Shafin *et al.*, 2020).

Both Racon and MarginPolish are polishers that make use of quality scores. Medaka and HELEN, on the other side, are designed to refine the base polish obtained by Racon and MarginPolish, respectively.

We tested each polishing pipeline on the original (non-quantized) data, and quantized versions using *F*_10_, *Q*_2_, *Q*_4_ and *Q*_8_. To account for randomness within the executed algorithms, we ran three executions of each combination of polisher and data set.

As a reference for the assessment of the results we used a combination of scaffolds obtained by SPAdes (Bankevich *et al.*, 2012) from Illumina reads as described in Nicholls *et al.* (2019) (http://nanopore.s3.climb.ac.uk/mockcommunity/v2/Zymo-Isolates-SPAdes-Illumina.fasta). We evaluated the quality of the final assembly for each run of a polishing pipeline on a data set using MetaQUAST (Mikheenko *et al.*, 2015) to obtain the number of mismatches per kilobase pairs (kbp) with respect to the combined reference.

To analyze the results of assembly polishing on quantized quality scores data in a different setting, we followed some of the experiments reported in Shafin *et al.* (2020) for human genome assembly. Specifically, we polished the assembly generated by wtdbg2 (Ruan and Li, 2020) for one of the flows for sample HG00733 (1000 Genomes Project Consortium *et al.*, 2015) with the polishing pipelines MP and Helen. These polishing pipelines were executed both for the original FASTQ files and for the same data quantized with *Q*_4_, and we compared the number of mismatches per 100 kbp with respect to the reference GRCh38 using QUAST (Gurevich *et al.*, 2013). We carried on this comparison for several coverage scenarios, which we obtained by randomly selecting a fraction (10%, 20%, 60% and 100%) of the dataset reads. We repeated each execution three times to account for algorithm randomization.

### 2.3 Variant calling

We compared the nanopore variant calling performance of PEPPER-Margin-DeepVariant on sample HG003, as reported in Shafin *et al.* (2021), against variant calling on quantized versions of the same data. We performed this comparison at various coverages, ranging from 20× to 90×, and for various quantizers described in Section 2.1.

Following (Shafin *et al.*, 2021), we used GIAB v4.2.1 truth set against GRCh38 reference, and GIAB stratification v2.0 files with hap.py (http://github.com/illumina/hap.py) to derive stratified variant calling results. As a result, for each coverage and each quantized version of the data, we obtain a classification of the variants reported by PEPPER-Margin-DeepVariant into two categories: true-positives (TP), and false-positives (FP), and a set of true variants that PEPPER-Margin-DeepVariant failed to identify, i.e. false-negatives (FN). These variants are reported separately for SNPs and insertions/deletions (INDELs). From these reports, we summarize the performance of PEPPER-Margin-DeepVariant for each specific type of variant (SNP/INDEL), on various coverage and quantization settings, by the *recall*, *precision* and F1*score*: $\text{Rec}=\frac{\text{TP}}{\text{TP}+\text{FN}},\;\text{Prec}=\frac{\text{TP}}{\text{TP}+\text{FP}},\;F1=\frac{2\times \text{Prec}\times \text{Rec}}{\text{Prec}+\text{Rec}}$.

### 2.4 Compressibility

To evaluate the improvement in compressibility obtained by quantizing the quality scores of nanopore FASTQ files, we used sample HG003 (the same data set used for variant calling evaluation), which consists of three FASTQ files that add up to ∼520 GB. We compressed the original data set and quantized versions of this data set using the general purpose compressor gzip (https://www.gnu.org/software/gzip/). For each evaluated quantizer, we calculated the *compression ratio (CR)*, defined as the quotient between the size of the compressed data set and the size of the original data set (smaller ratios correspond to better compression performance).

## 3 Results

Table 2 presents the number of mismatches per 100 kbp for the assembly of a mock microbial community with respect to a combined reference (see Section 2.2), for various combinations of assembly polishing algorithm and quality score quantizer. The number of mismatches, averaged over three independent runs, is very similar for all tested combinations of polisher and quantizer, with differences that are generally smaller than those between different runs of the same polisher and quantizer, which arise from algorithm randomization in the polishing pipeline. Comparing the average number of mismatches obtained with a certain polisher using quantized data and using the original data, we observe that the maximum difference is obtained for Medaka with the most heavy quantizer, *F*_10_. Even in this case, the difference represents only 1.2% of the average number of mismatches obtained with the original data. Notably, the results for the quantizer *Q*_4_ are even better than those obtained with the original data for all the evaluated polishing pipelines.

**Table 2.** Number of mismatches per 100 kbp for the assembly of a mock microbial community, with respect to a combined reference, for various combinations of assembly polishing algorithm and quality score quantizer

Polishing	Original		*Q*_8_		*Q*_4_		*Q*_2_		*F*_10_	
	Run	Avg.	Run	Avg.	Run	Avg.	Run	Avg.	Run	Avg.
Racon	180.59	179.46	180.05	179.35	177.93	177.54	179.74	179.09	180.07	179.21
	178.51		180.43		175.97		178.16		179.64	
	179.28		177.56		178.72		179.37		177.92	
Medaka	175.14	175.56	176.24	175.22	174.32	174.17	175.01	175.18	178.10	177.69
	174.97		175.78		173.20		175.56		178.01	
	176.56		173.63		175.00		174.97		176.95	
MP	175.57	176.26	176.02	175.94	176.47	174.95	175.41	176.26	174.70	175.23
	176.79		176.28		173.47		176.66		174.57	
	176.43		175.53		174.92		176.70		176.43	
Helen	167.14	167.75	168.03	167.92	168.17	167.31	168.53	168.48	167.29	167.92
	168.33		168.46		166.00		168.47		167.98	
	167.79		167.26		167.75		168.43		168.48	

*Note*: For each polishing algorithm and each quantizer, we show the result for three independent runs and the average of these three results.

Table 3 compares the number of mismatches per 100 kbp for a human genome assembly obtained with the original (non-quantized) data and the data obtained by applying *Q*_4_ to quality scores. The comparison is performed for the polishers MP and Helen for the full data set (100%), and for downsampled versions of the data consisting of 10%, 20% and 60% of the reads in the full data set (see Section 2.2). For a 20% or larger fraction of the data, the results obtained after quantization are essentially equivalent to those obtained with the original data. Even for a very small fraction of the data (10%), the percent increment in the number of mismatches incurred by quantization, averaged over three independent runs, is 0.01% for MP and 0.7% for Helen.

**Table 3.** Number of mismatches per 100 kbp obtained by MP and Helen for a human genome assembly, under various coverage scenarios, for the original (non-quantized) data and for the data obtained by applying *Q*_4_ to quality scores

Polishing	Quantization	10%		20%		60%		100%	
		Run	Avg.	Run	Avg.	Run	Avg.	Run	Avg.
MP	Original	1480.01	1480.01	935.24	935.24	132.89	132.89	100.32	100.32
		1480.01		935.24		132.89		100.32	
		1480.01		935.24		132.89		100.33	
	*Q*_4_	1480.01	1480.16	935.24	935.24	131.01	132.26	100.33	100.32
		1480.24		935.24		132.89		100.32	
		1480.24		935.24		132.89		100.32	
Helen	Original	1470.56	1460.03	907.05	907.05	125.94	125.94	99.65	99.65
		1454.77		907.05		125.94		99.65	
		1454.77		907.05		125.94		99.65	
	*Q*_4_	1470.56	1470.56	907.05	907.05	125.98	125.98	99.65	99.65
		1470.56		907.05		125.98		99.65	
		1470.56		907.05		125.98		99.65	

*Note*: For each combination of polisher, quantizer and coverage, we show the result for three independent runs and the average of these three results.

**Fig. 1.**
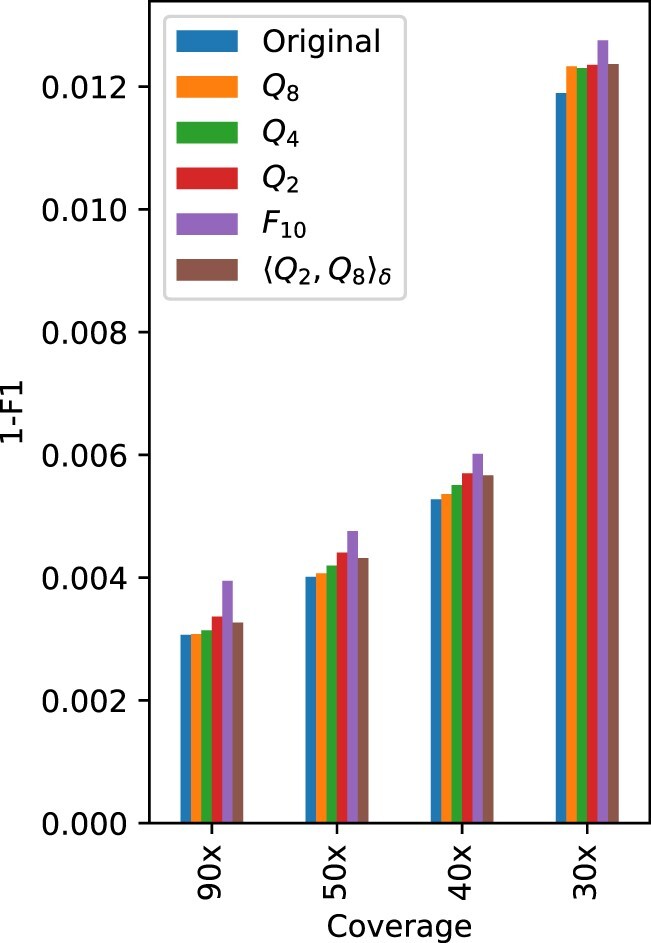
Plot of 1−F1 score for the SNPs obtained with PEPPER-Margin-DeepVariant for the original (non-quantized) data and for the data obtained by applying various quality score quantizers, for coverages 90×, 50×, 40× and 30×

**Fig. 2.**
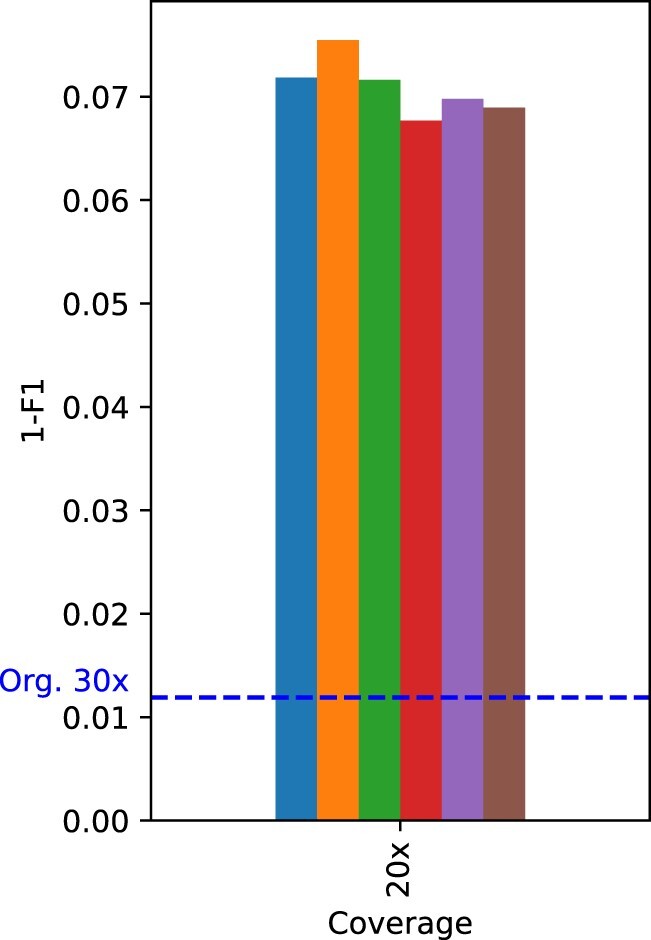
Plot of 1−F1 score for the SNPs obtained with PEPPER-Margin-DeepVariant for the original (non-quantized) data and for the data obtained by applying various quality score quantizers, for coverage 20×. As a reference, the dotted line marks the value 1−F1 for the original data with coverage 30×

**Fig. 3.**
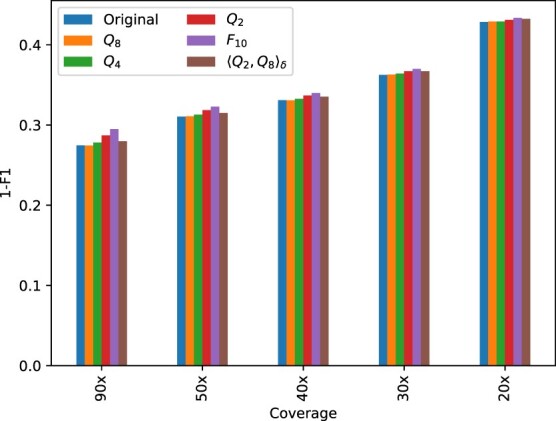
Plot of 1−F1 score for the INDELs obtained with PEPPER-Margin-DeepVariant under various coverage scenarios, for the original (non-quantized) data and for the data obtained by applying various quality score quantizers

Tables 4 and 5 show the count of true-positives, false-positives, and false-negatives for SNPs and INDELs, respectively, obtained with PEPPER-Margin-DeepVariant for various quantization schemes and coverage levels. The tables also show the metrics Rec, Prec and F1 calculated from these counts. The complement to 1 of the F1 score, i.e. the difference 1−F1, is shown for the same quantization schemes and coverage levels in Figures 1 and 2 for SNPs, and in Figure 3 for INDELs.

**Table 4.** Metrics TP, FP, FN, Rec, Prec and F1 for the SNPs obtained with PEPPER-Margin-DeepVariant under various coverage scenarios, for the original (non-quantized) data and for the data obtained by applying various quality score quantizers

Cov.	Quant.	TP	FP	FN	Rec	Prec	F1
90×	Original	3 317 116	10 041	10 376	0.9969	0.9970	0.9969
	*Q*_8_	3 317 064	10 071	10 428	0.9969	0.9970	0.9969
	*Q*_4_	3 316 599	9994	10 893	0.9967	0.9970	0.9969
	*Q*_2_	3 315 132	10 023	12 360	0.9963	0.9970	0.9966
	*F*_10_	3 310 775	9522	16 717	0.9950	0.9971	0.9961
	${\langle Q_{2},Q_{8}\rangle }_{\delta }$	3 315 867	10 121	11 625	0.9965	0.9970	0.9967
50×	Original	3 314 614	13 842	12 878	0.9961	0.9958	0.9960
	*Q*_8_	3 314 564	14 160	12 928	0.9961	0.9957	0.9959
	*Q*_4_	3 314 059	14 488	13 433	0.9960	0.9956	0.9958
	*Q*_2_	3 312 857	14 693	14 635	0.9956	0.9956	0.9956
	*F*_10_	3 310 166	14 337	17 326	0.9948	0.9957	0.9952
	${\langle Q_{2},Q_{8}\rangle }_{\delta }$	3 313 530	14 780	13 962	0.9958	0.9956	0.9957
40×	Original	3 312 510	20 159	14 982	0.9955	0.9940	0.9947
	*Q*_8_	3 312 554	20 778	14 938	0.9955	0.9938	0.9946
	*Q*_4_	3 312 056	21 237	15 436	0.9954	0.9936	0.9945
	*Q*_2_	3 310 979	21 442	16 513	0.9950	0.9936	0.9943
	*F*_10_	3 308 694	21 260	18 798	0.9944	0.9936	0.9940
	${\langle Q_{2},Q_{8}\rangle }_{\delta }$	3 311 573	21 820	15 919	0.9952	0.9935	0.9943
30×	Original	3 307 863	60 007	19 629	0.9941	0.9822	0.9881
	*Q*_8_	3 307 922	63 022	19 570	0.9941	0.9813	0.9877
	*Q*_4_	3 307 467	62 382	20 025	0.9940	0.9815	0.9877
	*Q*_2_	3 306 457	61 682	21 035	0.9937	0.9817	0.9876
	*F*_10_	3 303 993	61 874	23 499	0.9929	0.9816	0.9872
	${\langle Q_{2},Q_{8}\rangle }_{\delta }$	3 306 928	62 256	20 564	0.9938	0.9815	0.9876
20×	Original	3 285 668	466 868	41 824	0.9874	0.8756	0.9282
	*Q*_8_	3 285 516	494 441	41 976	0.9874	0.8692	0.9245
	*Q*_4_	3 284 787	464 226	42 705	0.9872	0.8762	0.9284
	*Q*_2_	3 283 171	432 505	44 321	0.9867	0.8836	0.9323
	*F*_10_	3 277 795	442 196	49 697	0.9851	0.8812	0.9302
	${\langle Q_{2},Q_{8}\rangle }_{\delta }$	3 283 622	442 501	43 870	0.9868	0.8813	0.9311

**Table 5.** Metrics TP, FP, FN, Rec, Prec and F1 for the INDELs obtained with PEPPER-Margin-DeepVariant under various coverage scenarios, for the original (non-quantized) data and for the data obtained by applying various quality score quantizers

Cov.	Quant.	TP	FP	FN	Rec	Prec	F1
90×	Original	303 517	29 403	200 983	0.6016	0.9136	0.7255
	*Q*_8_	303 713	29 607	200 787	0.6020	0.9131	0.7256
	*Q*_4_	301 600	30 180	202 900	0.5978	0.9110	0.7219
	*Q*_2_	296 238	30 968	208 262	0.5872	0.9074	0.7130
	*F*_10_	289 838	28 569	214 662	0.5745	0.9123	0.7050
	${\langle Q_{2},Q_{8}\rangle }_{\delta }$	304 333	37 142	200 167	0.6032	0.8935	0.7202
50×	Original	287 895	43 665	216 605	0.5707	0.8709	0.6895
	*Q*_8_	287 890	44 025	216 610	0.5706	0.8699	0.6892
	*Q*_4_	286 319	43 663	218 181	0.5675	0.8703	0.6870
	*Q*_2_	283 147	44 307	221 353	0.5612	0.8673	0.6815
	*F*_10_	279 418	42 311	225 082	0.5539	0.8711	0.6772
	${\langle Q_{2},Q_{8}\rangle }_{\delta }$	289 053	51 537	215 447	0.5729	0.8515	0.6850
40×	Original	278 841	51 239	225 659	0.5527	0.8476	0.6691
	*Q*_8_	278 920	51 314	225 580	0.5529	0.8475	0.6692
	*Q*_4_	277 724	51 171	226 776	0.5505	0.8473	0.6674
	*Q*_2_	275 492	51 998	229 008	0.5461	0.8442	0.6632
	*F*_10_	272 761	50 237	231 739	0.5407	0.8474	0.6601
	${\langle Q_{2},Q_{8}\rangle }_{\delta }$	280 046	59 427	224 454	0.5551	0.8281	0.6646
30×	Original	265 283	63 876	239 217	0.5258	0.8092	0.6374
	*Q*_8_	265 193	64 151	239 307	0.5257	0.8085	0.6371
	*Q*_4_	264 442	64 213	240 058	0.5242	0.8079	0.6358
	*Q*_2_	263 047	65 037	241 453	0.5214	0.8051	0.6329
	*F*_10_	260 681	63 727	243 819	0.5167	0.8069	0.6300
	${\langle Q_{2},Q_{8}\rangle }_{\delta }$	266 429	72 561	238 071	0.5281	0.7894	0.6329
20×	Original	239 522	96 040	264 978	0.4748	0.7177	0.5715
	*Q*_8_	239 532	96 766	264 968	0.4748	0.7162	0.5710
	*Q*_4_	238 938	95 403	265 562	0.4736	0.7185	0.5709
	*Q*_2_	237 475	94 862	267 025	0.4707	0.7185	0.5688
	*F*_10_	235 169	92 383	269 332	0.4661	0.7219	0.5665
	${\langle Q_{2},Q_{8}\rangle }_{\delta }$	239 968	102 876	264 532	0.4757	0.7039	0.5677

For SNPs, Table 4 shows high recall and precision values, specially for large coverage levels. In all cases, the difference between the F1 score obtained with the original data and that obtained with a quantized version of the same data is rather small. The largest difference for coverage 30× and above is smaller than $10^{-3}$ (for *F*_10_ on 90× data). For each of these coverage levels, the performance slightly improves in general with the quantization precision, and the performance for ${\langle Q_{2},Q_{8}\rangle }_{\delta }$ lies between that of *Q*_2_ and *Q*_4_ (see Fig. 1). For the shallowest coverage, 20×, the performance is noticeable worse (see Fig. 2 and note the different scale compared to Fig. 1). In this case, the F1 score does not always improve with the quantization precision. Following the performance comparison criteria in Shafin *et al.* (2021), we notice that PEPPER-Margin-DeepVariant on 90× data still outperforms DeepVariant on Illumina short reads (35× Illumina NovaSeq, F1:0.9963) for all quantizers except *F*_10_. Another interesting reference for comparison is the variation in performance obtained by using different variant calling pipelines. For example, using Longshot (Edge and Bansal, 2019) on the 30× original data we obtain a precision of 0.9648 and a recall of 0.9743, which determine an F1 score of 0.9695 for SNPs. Since Longshot ignores quality scores, the results after quality score quantization are exactly the same. Notice that these metric values fall below those reported in Table 4 for every quantizer on 30× data, including *F*_10_, which looses all quality score information from the original data. Thus, in this example, the choice of a variant calling pipeline causes larger performance variations than the quantization of quality scores.

For INDELs, in agreement with Shafin *et al.* (2021), the recall and precision values are significantly worse than those obtained for SNPs. The difference between the performance obtained with non-quantized and quantized data is still small, although more noticeable than the difference obtained for SNPs. For INDELs, the largest difference in F1 score occurs for *F*_10_ on 90× data, with a difference approximately equal to 0.002. For *Q*_4_, the maximum difference drops to $3.6\times 10^{-3}$, and for *Q*_8_ it is $4.7\times 10^{-4}$. The variant calling performance for the data quantized with ${\langle Q_{2},Q_{8}\rangle }_{\delta }$ lies, in general, between those obtained with *Q*_2_ and *Q*_4_.

Table 6 shows the CR obtained with gzip for various quantizers. The table also shows the space saving obtained by quality score quantization, calculated as the difference in size between the compressed original data set and the compressed quantized data set, expressed as a percent of the size of the compressed original data set. For example, the table shows that gzip compresses the original data set to roughly half the original size (the CR is 0.49), and *Q*_4_ improves the CR to 0.25, yielding a compressed data set that is roughly half the size of the compressed original data set (a saving of 48.52%). For the coarsest quantizer, *F*_10_, the difference in compressed size is almost 70% of the original compressed data set, which in the evaluated data set amounts to more than 175 GB of saved space. For easy assessment of the relation between CR and variant calling performance, Table 6 shows, for each quantizer, the F1 score obtained with quantized 90× coverage data.

**Table 6.** CR obtained with gzip for the original data and for various quantized versions of the same data

Quantization	CR	Space saving (%)	F1 score at 90×
Original	0.49	–	0.9969
*Q*_8_	0.32	33.7	0.9969
*Q*_4_	0.25	48.5	0.9969
*Q*_2_	0.19	60.7	0.9966
*F*_10_	0.15	69.3	0.9961
${\langle Q_{2},Q_{8}\rangle }_{\delta }$	0.22	54.9	0.9967

*Note*: The third column presents the percent space saving of the gzip compression of each quantized version compared to the original data compressed with gzip. The right-most column shows, for each quantization level, the F1 score obtained with coverage level 90×.

**Fig. 4.**
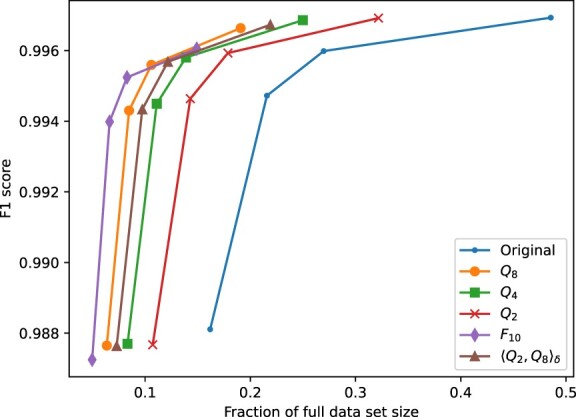
F1 score for the SNPs obtained with PEPPER-Margin-DeepVariant as a function of the required storage space, for each evaluated quantizer and coverage levels 90×, 50×, 40× and 30×. The storage requirement is expressed as a fraction of the size of the full (uncompressed, 90×) data set

The tradeoff between storage space and variant calling performance is also illustrated in Figure 4. For each quantizer and coverage level ℓ (excluding 20×), we multiply the gzip CR for that quantizer by the coverage level ratio, ℓ/90. This value represents the fraction of storage space required by the data with coverage level ℓ, quantized and compressed with gzip, with respect to the full (uncompressed, 90×) data set. These fractions of storage space are plotted in the figure against the F1 score obtained for SNPs in each case. Notice, for example, that higher coverage data quantized with *Q*_4_ yields better performance than lower coverage non-quantized data (except at 90× coverage), while requiring actually less storage space.

## 4 Conclusions

Our experiments on various usage scenarios for nanopore sequencing data, including different applications and coverage levels, show that the precision that is currently used for quality scores may be unnecessarily high. A quantizer like *Q*_4_, which uses only four different values for quality scores, yields results similar to those obtained with the original data in all tested scenarios. We point out that all these results were obtained with applications *as they are provided*, with no special tuning or training for quantized quality scores, nor with any quantizer optimization. Although such specific application tuning may improve the performance of these applications (for example through neural network retraining), the fact is that excellent results are obtained with no software adjustment and no quantizer optimization. The quantization of quality scores results in large storage space savings, even using a general purpose compressor, such as gzip.

## Data Availability

The datasets used in this article were obtained from sources in the public domain as detailed in Section 2.
